# Law Demands “Care” - Not “Cure”

**DOI:** 10.4103/0974-1216.71610

**Published:** 2009

**Authors:** Gopinath Shenoy

**Affiliations:** Obstetrician-Gynaecologist and Medico Legal Consultant, Consumer Courts, Medical Councils, India

**Keywords:** Gynecological endoscopy, law, cure, care

## Abstract

In today’s medicolegal scenario specially related to endoscopic surgery, patient expects a miraculous result or a definite cure. However the author who has experience of both the fields i.e. gynecological endoscopy and medicolegal aspects, clears that the law doesn’t expect a 100% cure but expects proper medical care by the concerned gynecologist, gynecological endoscopist. Patient also has to be informed about this important fact.

## INTRODUCTION

The duty cast on a medical practitioner is based on the fact that he is treating a human being and his treatment can possibly cause physical damage to his patient unless he takes proper care and employs good skill.

A physician who diagnoses and treats a person for a disease or a surgeon who performs an operation on a patient to remove or rectify a defect, is for all practical purposes giving a warranty that he possesses the required skill and knowledge for that purpose.

He is duty bound in two respects, that is, he owes a primary duty of care in deciding whether he should undertake the case and after having undertaken the case, the next duty that is cast on him is the duty of care in the administration of that treatment wherein he should use due diligence, care and caution.

His failure to perform either of the above two duties, if proved, will offer reasonable and valid grounds to fasten the charge of negligence on him. Law does not require him to possess the highest or a very high standard of care nor does the law require him to exhibit a very low standard.

From him, law expects only fair and reasonable standard of care and competence. Chief Justice Hewart has laid down this norm in an English case. He says

“*A doctor has to evince reasonable degree of skill and knowledge and must exercise a reasonable degree of care while practicing his profession. He cannot be expected to apply the ideal or the highest degree of skill and care while handling a case*”.

Justice Tindal, another English judge, adds

“*Every person who enters into a learned profession undertakes to bring to the exercise of it, a reasonable degree of care. He does not undertake, if he is an attorney, that at all events you shall gain your case, nor does a surgeon undertake that he will perform a cure, nor does he undertake to use the highest possible degree of skill. There may be persons who have higher education and greater advantages than he has, but he undertakes to bring a fair, reasonable and competent degree of skill*”.

The observation of the same judge in another case further elucidates this point thus:

“*A surgeon does not become an actual insurer, he is only bound to display sufficient skill and knowledge in his profession. If from some accident or some variation in the frame of a particular individual an injury happens, it is not a fault in the medical man*”.

In “*Bolam v Friern Hospital Management Committee*” Justice McNair observed:

“*A man need not possess the highest expert skill; it is well established law that it is sufficient if he exercises the ordinary skill of an ordinary competent man exercising that particular art*”.

Lord Justice Clyde in another English case observes

“*The true test of establishing negligence in diagnosis or treatment on the part of a doctor is whether he has been proved to be guilty of such failure as no doctor of ordinary skill would be guilty of, if acting with ordinary care*.”

Lord President Clyde in Hunter v. Hanley [(1955) SLT 213] further holds:

“*The medical man must, therefore, exercise reasonable skill and care, measured by the standard of what is reasonably to be expected from the ordinary competent practitioner of his class. If he does so he will have discharged his duty and cannot be held answerable even if the treatment has untoward results. For the medical man is not an insurer; he does not warrant that his treatment will succeed or that he will perform a cure. Naturally he will not be liable if, by reason of some peculiarity in the frame or constitution of a patient which was not reasonably to be anticipated a treatment in ordinary circumstances would be sound, has unforeseen results. But he will not even be liable for every slip of accident*.”

The various High Courts and the Supreme Court of India have been generally following the principles and norms discussed above in deciding cases of medical negligence.

The Hon’ble Supreme Court in Jacob Mathew Petitioner v. State of Punjab and Anr. Respondent 2005(3) CPR 70 (SC) holds:

“*He does not assure his client of the result. A lawyer does not tell his client that the client shall win the case in all circumstances. A physician would not assure the patient of full recovery in every case. A surgeon cannot and does not guarantee that the result of surgery would invariably be beneficial, much less to the extent of 100% for the person operated on. The only assurance which such a professional can give or can be understood to have given by implication is that he is possessed of the requisite skill in that branch of profession which he is practicing and while undertaking the performance of the task entrusted to him he would be exercising his skill with reasonable competence. This is all what the person approaching the professional can expect*.”

In determining whether a doctor has exercised a reasonable skill and care, due regard must be given to the general and approved practices of the profession or the accepted practices followed by other practitioners of similar status.

Here two general principles may be stated with confidence:

1. If a medical practitioner acts in accordance with the general and approved practice of the profession, he cannot be held to be negligent unless the exceptional circumstances of the case warrant such a finding.
2. Equally important is the fact that if a doctor departs from the general and approved practice for no good reason resulting in damage and injury to the patient, he is likely to be held negligent.

These are evidently matters of facts and necessarily involve receiving expert evidence. The value of expert evidence is obvious in a case where negligence is alleged against a medical man or a medical institution. It may be remembered that the cardinal test for determining the question of medical negligence is whether a reasonably competent medical man would have acted in more or less the same manner in which the medical man against whom negligence is alleged had acted. It is therefore natural that courts of law will appreciate the assistance of expert medical evidence to ascertain this point.

The above discussion should not be viewed or misconstrued as an unjustifiable opening and easy exit for delinquent doctors to escape from liability due to negligence. Misrepresenting that one possesses the skill or expertise which he does not or recklessness in undertaking a treatment or recklessness in the conduct of it or indifferent handling of cases or failure to act diligently and alertly at the appropriate time or evident negligence like amputating a wrong limb or administering a prohibited or known counterproductive medicine or wrong diagnosis or treatment which under no norms of general or approved practice can be justified, will certainly land a medical practitioner in difficulties and expose him to liability for negligence.

Caution is therefore required whilst appreciating evidence and applying the law in complaints involving negligence of doctors. The findings and decision in cases of medical negligence should be made with utmost caution such that it does not result in a professional nightmare to the new entrants into this field of noble service to humanity or drive away really sincere and dedicated practitioners from the field.

